# Knowledge and practice of pregnant women and health care workers on hepatitis B prevention in the Limbe and Muyuka health districts of the south west region of Cameroon

**DOI:** 10.11604/pamj.2019.33.310.16894

**Published:** 2019-08-20

**Authors:** Brenda Mbouamba Yankam, Cho Sebastine Anye, Ngwayu Claude Nkfusai, Joyce Shirinde, Samuel Nambile Cumber

**Affiliations:** 1 Department of Microbiology and Parasitology, Faculty of Science, University of Buea, Buea, Cameroon; 2 Department of Statistics, Faculty of Physical Science, University of Nsukka, Nsukka, Nigeria; 3 Cameroon Baptist Convention Health Services (CBCHS), Yaoundé, Cameroon; 4 School of Health Systems and Public Health, Faculty of Health Sciences, University of Pretoria Private Bag X323, Gezina, 0001, Pretoria, South Africa; 5 Section for Epidemiology and Social Medicine, Department of Public Health, Institute of Medicine, The Sahlgrenska Academy at University of Gothenburg, Box 414, SE - 405 Gothenburg, Sweden; 6 Faculty of Health Sciences, University of the Free State, Bloemfontein, South Africa

**Keywords:** Hepatitis B virus, pregnant women, health care workers, risk factors, knowledge, practice, south west region, Cameroon

## Abstract

**Introduction:**

Hepatitis B virus (HBV) infection is a major health problem worldwide owing to its high prevalence and significant morbidity and mortality. There are about 2 billion people living with HBV worldwide and over 360 million chronic carriers. The purpose of this study was to assess the knowledge and practices of pregnant women and health care workers in the ANC and maternity units on HBV infection and its transmission.

**Methods:**

About 270 women attending ANC and 31 health care workers were selected by convenience sampling. They were evaluated using a structured questionnaire to assess their knowledge and practices on HBV prevention and transmission.

**Results:**

Pregnant women in the Limbe Health District demonstrated good knowledge but adopted poor practices whereas in the Muyuka Health District, pregnant women demonstrated poor knowledge and adopted poor practices regarding the mode of transmission and prevention of HBV infection. Health care workers in both the Limbe and Muyuka Health Districts however, demonstrated good knowledge and adopted good practices.

**Conclusion:**

There was a significant relationship between the knowledge and practice of pregnant women and health care workers on Hepatitis B prevention in the Muyuka Health District (P = 0.0006).

## Introduction

Hepatitis B Virus (HBV) is a small enveloped DNA virus known to infect humans and belongs to the hepadnaviridae family. HBV infection is a major health problem worldwide owing to its high prevalence and significant morbidity and mortality. There are about 2 billion people living with HBV worldwide and about 360 million chronic carriers [1]. The total number of deaths attributable to HBV was 786,000 according to the Global Burden of Disease (GBD), 2010. Of these number, 132,200 (17%) were estimated to be caused by acute hepatitis B, 341,400 (43%) were caused by liver cancer and 312,400 (40%) were caused by cirrhosis [2]. As a result, the GBD 2010 estimates HBV to be the 15^th^ ranked caused of human death [2]. It is transmitted through sexual intercourse, by exchange of saliva during kissing and also from infected mothers to their babies: during childbirth, breastfeeding and through the placenta [3]. Unlike Human Immunodeficiency Virus (HIV), the hepatitis B virus can survive outside the body for at least seven days [4]. During this time, the virus can still cause infection if it enters the body of someone who is not protected by the vaccine. The burden of HBV is highest in Africa where approximately 65 million chronically infected individuals live, with prevalence rates ranging from less than 7% to more than 20% in some countries [5]. Recent studies in Cameroon show that the prevalence of HBV ranges from 6-16% [6, 7]. Fouelifack [8], Noubiap [7] report the prevalence of HBV as high as 10.1% and 12% among blood donors in hospital blood banks in Cameroon respectively. Pregnant women are considered vulnerable and even at a higher risk of transmitting the virus to their newborns if not early diagnosed. As far as the burden of HBV in health care workers is concerned, numerous studies have shown that they are at higher risk of acquiring HBV than the general population. Clinicians with direct patient contact, such as physicians, dentists, nurses, and dialysis workers, laboratory workers have higher risk of exposure to HBV than other health care workers [9]. A review of studies done in the USA has shown a high prevalence rate of HBV ranging from 13 to 18% in some groups of health care workers such as surgeons, [10]. In sub-Saharan Africa too, exposure to HBV remains a serious risk to health care workers. It has been estimated that 6,200 HBV infections occur each year among health care workers in sub-Saharan African [11]. This study was therefore designed to assess the knowledge and practice of pregnant women attending antenatal clinic (ANC) and health care workers at the ANC and maternity units in the Limbe and Muyuka health districts of the south west region of Cameroon, with specific objective to determine the risk factors of hepatitis B virus infection among pregnant women and health care workers in the Limbe and Muyuka health districts.

## Methods

**Study area:** Limbe is the capital of Fako Division. It has a total population of 72,106 inhabitants as of 2015 [12]. It is located on latitude 40’46.008” N, longitude 913’13.008” E and about 15 km from Buea, the headquarters of the south west region of Cameroon. Muyuka is a small town in Fako Division of the south west region of Cameroon, it is located on latitude 417’49.992” N, longitude 924’20.016” E and about 30.6 km from Buea. It is made up of 18 villages with about 31,384 inhabitants as of 2015 [12].

**Study sites:** this study was conducted at the Regional Hospital Limbe, District Hospital Limbe, District Hospital Muyuka and Presbyterian Health Center Muyuka of the south west region of Cameroon.

**Study design:** the study involved administration of questionnaire to pregnant women attending ANC and health care workers in the ANC and maternity units. A convenience sampling technique was employed whereby pregnant women attending ANC and health care workers in the ANC and maternity units available at the time of the study and who were willing to participate in the study were included for the purpose of the study.

**Study population:** the study population constituted of pregnant women attending ANC and health care workers in the ANC and maternity units in all the hospitals under study who gave consent to take part in the study from July to August 2017.

### Selection criteria

**Inclusion criterion:** pregnant women attending ANC and health care workers at the ANC and maternity units present during the period of data collection (July-August) and who gave consent to participate in the study.

**Exclusion criteria:** pregnant women and health care workers who did not consent to participate in the study.

**Sample size and sampling:** the sample size for questionnaire administration to the pregnant women was calculated using the Fisher’s formula, with a prevalence of 9.7% of hepatitis B infection [6] among pregnant women with an error margin (d) of 0.05.

$${\text{N=Z}}^{\text{2}}{\text{*P(1-P)/d}}^{\text{2}}$$

Where: z = the standard normal deviation at 1.96 (which corresponds to a 95% confidence interval), p = the prevalence of hepatitis B in the general Cameroonian population, q = 1 – p; d = the degree of precision expected to be 0.05. Therefore, N= 1.96²*0.097 (1-0.097)/0.05²; N = 135.

Therefore, sample size for questionnaire administration to pregnant women was 135 questionnaires per health district giving a total of 270 questionnaires. The sample size for questionnaire administered to health care workers was exhaustive, that is, all health care workers at the ANC and maternity unit of the study sites who consented to respond to the questionnaires were included.

### Data collection

#### Administration of questionnaires to pregnant women and health care workers

All participants who consented were interviewed using a structured questionnaire adapted from questionnaire formulated by Mohammed [13]. Prior to its use in this study, a total of 20 questionnaires were pretested at the Regional Hospital Buea among pregnant women attending ANC and health care workers at the ANC and maternity unit with the aim of revising poorly structured questions, estimate the average time required to fill the questionnaire. Two hundred and seventy questionnaires were administered to pregnant women attending ANC and 31 questionnaires were administered to health care workers at the ANC and maternity in all the hospitals under study for a period of 2 months (July-August) to assess their knowledge and practices on hepatitis B prevention and transmission.

Knowledge on HBV infection consisted of 12 questions and each correct response was scored as 1 and 0 for a wrong response. The knowledge scores for an individual was calculated and summed up to give a total knowledge score on 12. A score between 0-4 was classified as poor, 5-8 as good and 9-12 as excellent adapted from a study conducted by Abongwa [14]. Practices of pregnant women and health care workers on HBV infection were assessed on a scale of 6 because there were 6 questions on practices regarding HBV infection. A score of 0-3 was classified as poor practice while a score of 4-6 was classified as good practice. Demographic information of the participant was also obtained through administration of questionnaires.

#### Data entry and analyses

Data from the questionnaires were entered into a template in Excel version 10. The data was verified for completion, cleaned and exported into SPSS v 20.0 for analyses. Descriptive analysis was carried out by calculating the mean, median, standard deviation and frequencies of different variables using the SPSS v 20.0.

#### Ethical considerations

Ethical clearance was obtained from the Institutional Review Board of the Faculty of Health Science, University of Buea. Administrative clearance was obtained from the regional delegation of public health for south west region Cameroon and written approval from the head of every hospital under study. Participants had the study protocol carefully explained to them and participation was voluntary. Written informed consent was obtained from all participants. Study participants, data confidentiality and integrity were maintained by restricting access of the information and primary data to the principal investigator.

## Results

### Socio-demographic characteristics of pregnant women in the Limbe and Muyuka health districts

The characteristics of the 270 pregnant women who responded to the questionnaires in the Limbe and Muyuka health districts are summarized on Table 1. The ages of these women ranged from 16 to 46 years with a mean ± SD age of 22.6 ± 5.6 years, with the predominant age group being pregnant women of 25-34 years. About 78 (28.9%) of the pregnant women were students, 119 (44.1%) were polygamously married, 8 (3.0%) had multiple sexual partners and only 13 (4.8%) of them were Muslims (Table 1).

**Table 1:** demographic characteristics of the 270 pregnant women attending antenatal clinic in the Limbe and Muyuka Health Districts, July-August 2017

Characteristics (n=270)	Stratification	Frequency	Percentage %
Age	<25	116	43.0
	25-34	130	48.1
	˃35	24	8.9
	Total	270	100
Occupation	civil servant	49	18.2
	Jobless	67	24.8
	Self employed	76	28.1
	Student	78	28.9
	Total	270	100
Area of residence	Urban	133	49.3
	Rural	137	50.7
	Total	270	100
Marital status	Single	121	44.8
	Married monogamy	119	44.1
	Married polygamy	22	8.1
	Divorced	3	1.1
	Widow	5	1.9
	Total	270	100
Parity	First pregnancy	107	39.6
	Primipara (1)	60	22.2
	Multipara (2–4)	102	37.8
	Grand multipara (>4)	1	0.4
	Total	270	100
Sexual Partner	Single	262	97.0
	Multiple	8	3.0
	Total	270	100
Religion	Christian	257	95.2
	Muslim	13	4.8
	Total	270	100

The characteristics of the 31 health care workers who responded to the questionnaires in the Limbe and Muyuka health districts are summarized on Table 2. The ages of these health care workers ranged from 20 to 48 years with a mean ± SD age of 22.6 ± 5.6 years, with the predominant age group being health care workers of < 25 years. About 21 (67.7%) of the participants were female, 16 (51.6 %) were state register nurses, 6 (19.4%) had worked for the past three years (Table 2).

**Table 2:** demographic characteristics of health care workers at the ANC and maternity units in the Limbe and Muyuka Health Districts between 2014 and 2016

Characteristics (n=270)	Stratification	Frequency	Percentage %
Gender	Male	10	32.3
	Female	21	67.7
	Total	31	100
Age	<25	14	45.2
	25-34	10	32.3
	˃35	7	22.6
	Total	31	100
Level of education	Secondary school	3	9.7
	High school	12	38.7
	University	16	51.6
	Total	31	100
Level of training of the participant	Nurse	24	77.4
	State nurse	4	12.9
	Medical student	3	9.7
	Total	31	100
Number of years worked	One year	14	45.2
	Two years	5	16.1
	Three years	6	19.4
	Four years	3	9.7
	Five years	3	9.7

### Risk factors associated to HBV infection

Out of the 270 women who responded to the questionnaires, 257 (95.2%) had a history of ear piercing, 51 (18.9%) had a history of dental procedure, 20 (7.4%) had a history of blood transfusion and 52 (19.3%) had a history of surgical procedure meanwhile only 1 (0.4%) had a history of unsafe injection (Table 3).

**Table 3:** responses on risk factors to Hepatitis B infection among pregnant women attending antenatal care in the Limbe and Muyuka Health Districts

Risk factor	Stratification	Number (n=270)	Percentage (%)
Have you ever been transfused blood?	No	250	92.6
	Yes	20	7.4
	Total	270	100
Have you had any history of surgical procedure?	No	218	80.7
	Yes	52	19.3
	Total	270	100
Any history of dental procedure?	No	219	81.1
	Yes	51	18.9
	Total	270	100
Do you have any tattoo on your body?	No	266	98.5
	Yes	4	1.5
	Total	270	100
Have you ever had a history of unsafe injection?	No	269	99.6
	Yes	1	0.4
	Total	270	100
Have you ever under gone Caesarean section procedure?	No	246	91.1
	Yes	24	8.9
	Total	270	100
Any history of ear piercing?	No	13	4.8
	Yes	257	95.2
	Total	270	100
Risk factor	Stratification	Number (n=270)	Percentage (%)
Any history of ear piercing?	No	13	4.8
	Yes	257	95.2
	Total	270	100
Have you ever had liver problem or jaundice?	No	264	97.8
	Yes	6	2.2
	Total	270	100
Any history of Abortion or Miscarriage?	No	231	85.6
	Yes	39	14.4
	Total	270	100
Any history of Female circumcision?	No	267	98.9
	Yes	3	1.1
	Total	270	100
Have you ever been transfused blood?	No	250	92.6
	Yes	20	7.4
	Total	270	100
Have you had any history of surgical procedure?	No	218	80.7
	Yes	52	19.3
	Total	270	100
Any history of dental procedure?	No	219	81.1
	Yes	51	18.9
	Total	270	100

### Districts

Out of the 135 pregnant women who responded to the questionnaires in the Limbe Health District, 68 (50.45%) demonstrated excellent knowledge, 40 (29.85%) had good knowledge and 27 (19.69%) had poor knowledge on the transmission and prevention of hepatitis B virus infection. Regarding practices of pregnant women on transmission and prevention of Hepatitis B, 50 (37%) of them were classified as those adopting good practices in the transmission and prevention of Hepatitis B virus infection and 85 (63%) as those adopting poor practices.

**Figure 1:**
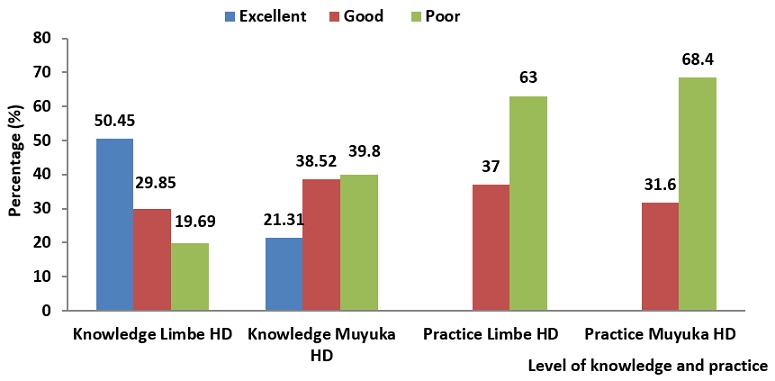
knowledge and practice of pregnant women in the Limbe and Muyuka health districts

Of the 135 women who responded to the questionnaires in the Muyuka Health District, 29 (21.31%) demonstrated excellent knowledge, 52 (38.52%) had good knowledge and 54 (39.8%) had poor knowledge on the transmission and prevention of hepatitis B virus infection. Regarding practices of pregnant women on transmission and prevention of hepatitis B, 43 (31.6%) of women were classified as adopting good practices and 92 (68.4%) as having poor practices (Figure 1).

Out of the 21 health care workers who responded to the questionnaires in the Limbe Health District, 16 (77.6%) demonstrated excellent knowledge, 2 (8.71%) had good knowledge and 3 (13.69%) had poor knowledge on the transmission and prevention of hepatitis B virus infection. Regarding practices of health care workers on the transmission and prevention of hepatitis B virus infection, 12 (55.83%) adopted good practices and 9 (44.17%) poor practices in the transmission and prevention of hepatitis B virus infection.

**Figure 2:**
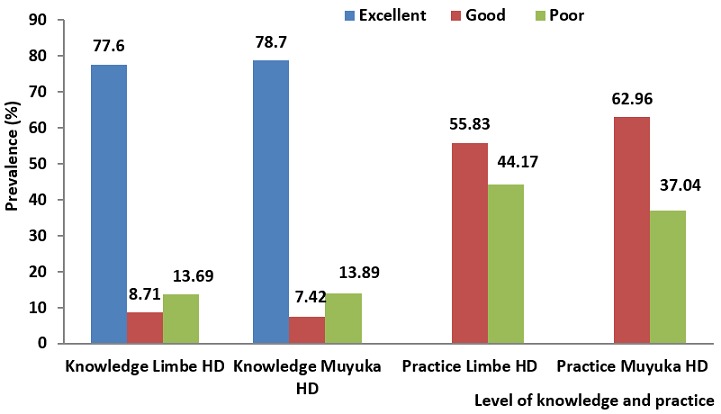
knowledge and practice of health care workers in the Limbe and Muyuka health districts

Of the 10 health care workers who responded to the questionnaires in the Muyuka Health District, 7 (78.7%) demonstrated excellent knowledge, 1 (7.42%) had good knowledge and 2 (13.89%) had poor knowledge on the transmission and prevention of hepatitis B virus infection while 6 (62.96%) adopted good practices (Figure 2).

The relationship between the knowledge and practice of pregnant women and health care workers on HBV prevention in the Limbe and Muyuka health districts is presented on the Table 4 and Table 5. There was a significant relationship between the knowledge and practice of pregnant women and health care workers on HBV infection in the Muyuka Health District with a P value of 0.0006 (Table 5).

**Table 4:** relationship between knowledge and practice of pregnant women and health care workers on HBV infection in the Limbe Health District

Variables LHD	Knowledge					Practice			
	Excellent	Good	Poor	Total	P value	Good	Poor	Total	P value
Pregnant women	68 (50.45%)	40 (29.85 %)	27 (19.69 %)	135 (100)	0.0711	50 (37 %)	85 (63 %)	135 (100)	0.0799
Health care workers	16 (77.6%)	2 (8.71%)	3 (13.69 %)	21 (100)		12 (55.83 %)	9 (44.17 %)	21 (100)	
Total	84	42	30	156		62	94	156	

**Table 5:** relationship between knowledge and practice of pregnant women and health care workers on HBV infection in the Muyuka Health District

Variables MHD	Knowledge					Practice			
	Excellent	Good	Poor	Total	P value	Good	Poor	Total	P value
Pregnant women	29 (21.31 %)	52 (38.52 %)	54 (39.8 %)	135 (100)	0.0006	43 (31.6 %)	92 (68.4 %)	135 (100)	0.0694
Health care workers	7 (78.7 %)	1 (7.42 %)	2 (13.89 %)	10 (100)		6 (62.96 %)	4 (37.04)	10 (100)	
Total	30	53	56	145		49	96	145	

## Discussion

We assessed the knowledge and practice of pregnant women and health care workers in the Limbe and Muyuka Health Districts of the South West Region of Cameroon. Data gathered in this work may provide more information to influence policy makers on the prevention and control of hepatitis B infection in pregnant women and health care workers. Pregnant women in the Limbe Health District demonstrated good knowledge but adopted poor practice regarding the mode of transmission and prevention of hepatitis B virus infection. Good knowledge of pregnant women regarding the mode of transmission and prevention of hepatitis B virus infection seen in our study is similar to studies reported in Egypt [15] and Japan [16]. Good knowledge of pregnant women in the Limbe Health District regarding the mode of transmission and prevention of hepatitis B virus infection can be explained by the fact that these women have been receiving regular ANC talks on what hepatitis B infection is.

On the contrary, in the Muyuka Health District, pregnant women demonstrated poor knowledge but adopted poor practices regarding the mode of transmission and prevention of hepatitis B virus infection. This is similar to the observation of Abongwa [14] and Frambo [6] where pregnant women demonstrated poor knowledge and adopted poor practices regarding the mode of transmission and prevention of hepatitis B virus infection in the north west region of Cameroon and Buea Health District respectively. Our observation is also similar to those reported in Ethopia [17]. The poor knowledge of pregnant women regarding the transmission and prevention of HBV in the Muyuka Health District can be explained by the lack of formal education among the pregnant women, many of whom come from the rural villages. This poor knowledge warrants the need for sustained and continuous education about hepatitis B virus infection.

In the Limbe and Muyuka health districts, health care workers demonstrated good to excellent knowledge regarding the mode of transmission and prevention of hepatitis B virus infection and adopted good practices regarding the mode of transmission and prevention of hepatitis B virus infection. Good knowledge and practice of health care workers in our study is similar to studies in Egypt [15] and Japan [16] but however contradict those reported by Yonatan [17] in Ethiopia and Kabir [18] in Iran. Good knowledge and practice of health care workers can be explained by the fact that they are medical personnel and their level of education is above primary and secondary school levels.

**Some limitations in our study include:** studying self-reported knowledge and practices is itself a limitation as one cannot rely totally on the information provided by the participants because of recall bias and social desirability bias.

## Conclusion

This study assessed the knowledge and practice of pregnant women and health care workers on the prevention and transmission of HBV infection in the Limbe and Muyuka health districts. Findings of our study indicate good knowledge (50.45%) and poor practice (63%) of pregnant women on hepatitis B infection in the Limbe Health District. It also indicates poor knowledge (39.8%) and poor practice (68.4%) of pregnant women on hepatitis B infection in the Muyuka Health District. Health care workers demonstrated good knowledge and good practice on hepatitis B infection in the Limbe and Muyuka Health District. There was a significant relationship between the knowledge and practice of pregnant women and health care workers on HBV infection in the Muyuka Health District (P = 0.0006).

### What is known about this topic

- In sub-Saharan Africa, exposure to HBV remains a serious risk to health care workers. It has been estimated that 6,200 HBV infections occur each year among health care workers in sub-Saharan African;
- Studies in the Buea Health District of the South West Region of Cameroon, show that about 80% of the pregnant women demonstrated poor knowledge regarding HBV prevention and transmission;
- A study carried out in Abakaliki Nigeria among pregnant women, show that 286 (71.5%) were aware of its occurrence in pregnancy while only 99 (24.8%) knew that hepatitis is a viral infection affecting the liver.

### What this study adds

- About 77% and 78% of health care workers in the Limbe and Muyuka health districts demonstrated excellent knowledge regarding HBV prevention and transmission;
- In the Limbe Health District 37% of pregnant women had good practices regarding HBV prevention and transmission meanwhile 31.6% had good practices regarding HBV prevention and transmission in the Muyuka Health District;
- About 55% and 62% of health care workers in the Limbe and Muyuka Health Districts had good practice regarding HBV prevention and transmission.

## Competing interests

The authors declare no competing interests.
